# Efficacy of neomycin dosing regimens for treating enterotoxigenic *Escherichia coli*-related post-weaning diarrhoea in a Danish nursery pig herd not using medicinal zinc oxide

**DOI:** 10.1186/s40813-022-00283-w

**Published:** 2022-11-04

**Authors:** Malene Kjelin Morsing, Inge Larsen, Ken Steen Pedersen, Nicolai Rosager Weber, Jens Peter Nielsen

**Affiliations:** 1 Department of Veterinary and Animal Sciences, University of Copenhagen, Grønnegårdsvej 2, 1870 Frederiksberg C, Denmark; 2 Danish Agriculture & Food Council, Axeltorv 3, 1609 Copenhagen V, Denmark

**Keywords:** Post-weaning diarrhoea, Medicinal zinc oxide, Neomycin, Antimicrobial resistance

## Abstract

Neomycin is a concentration-dependant aminoglycoside antimicrobial used to treat enterotoxigenic Escherichia *coli* (ETEC)-related post-weaning diarrhoea (PWD) in pigs. The objective was to compare the efficacy of neomycin administered in a single high dose (50,000 IU/kg) and a standard dose and frequency (25,000 IU/kg daily for 3 consecutive days) in reducing the number of pigs with clinical PWD. We also aimed to evaluate the development of antimicrobial resistance in *E. coli* following neomycin treatment. The study was performed in a Danish herd not using medicinal zinc oxide and experiencing outbreaks of PWD caused by ETEC in the first week after weaning. Pigs from six batches with perianal faecal staining on days 4–6 after weaning and a faecal score of 3–4 were ear tagged and treated with neomycin. Pens were randomly assigned to a treatment group before inclusion. A total of 772 pigs (471 in the control group and 301 in the experimental group) were included and treated orally. The apparent prevalence of diarrhoea on the first day of inclusion across six batches (n = 1,875) was 27%. The efficacy of the neomycin treatment strategy was 86% for the control group and 91% for the single high-dose group (*p* = *0.043*), and the mean percentage (standard deviation (sd)) of haemolytic *E. coli*-like colonies was 12% (26) and 26% (37) (*p* < *0.001*), respectively. Neomycin resistance did not differ between groups. Before treatment, all analysed isolates were identified as ETEC (n = 142), while after treatment, 91% were identified as ETEC (n = 69) and 9% (n = 7) as non-ETEC *E. coli* (without fimbria or toxins). A higher cure rate in the single high-dose group suggests that ETEC-related PWD can be treated with a single high dose of 50,000 IU/kg of neomycin, thereby reducing antimicrobial use by 33% compared to the standard treatment of 25,000 IU/kg for 3 consecutive days. The study indicated a higher number of haemolytic *E. coli* in the single high-dose group after treatment, but no evidence of increased neomycin resistance in coliforms was observed compared to the standard treatment.

## Introduction

Neomycin is a concentration-dependant aminoglycoside antimicrobial used to treat enterotoxigenic *E. coli* (ETEC)-related post-weaning diarrhoea (PWD) in pigs. In Denmark, the dosage is 25,000 IU/kg bodyweight (corresponding to 33 mg/kg) for 3–4 consecutive days, and batch medication via water is the most common administration route [1].

In 2018, the Danish Veterinary and Food Administration published a guide on antimicrobial use in pigs, with neomycin listed as the drug of choice for treating enteric colibacillosis, preferred over other aminoglycosides such as apramycin and gentamycin [2]. However, pharmacokinetic information on neomycin use in swine is very limited, and because of its concentration-dependant properties, it would be relevant to investigate the effect of an increased dose and decreased frequency compared to the standard neomycin treatment for pigs with ETEC-related PWD. Neomycin is poorly absorbed after oral administration, is to a large extent excreted in the faeces (> 90%) and remains primarily as the parent compound. A recent study showed that the neomycin elimination half-life in pigs after oral administration is long (12.43 ± 7.63 h at 15 mg/kg bodyweight) and the absolute bioavailability is low [3]. By using a single high dose of 50,000 IU/kg bodyweight, we can utilise the pharmacokinetic properties of the drug while also reducing the total amount of neomycin used to treat each pig. A further reduction in antimicrobial use can be achieved by implementing individual treatments as opposed to batch treatment. Nephrotoxicity and ototoxicity are among the possible critical adverse effects of a high dose or chronic use of aminoglycosides, yet this primarily applies to parental use or oral use in neonatal animals, where the bioavailability may be higher compared to older animals [4, 5].

There has been a slight decrease in the percentage of neomycin-susceptible haemolytic *E. coli* isolates from the intestinal tract in pigs (random clinical cases) in Denmark in recent years. In the first 6 months of 2021, neomycin susceptibility was 77% compared to 89–91% in 2014 [6]. Several studies have reported a relatively low prevalence of neomycin resistance in *E. coli* from pigs [7, 8], and a low number of neomycin-resistant *E. coli* isolates from finisher pigs (0.7% across three conventional herds) was seen in New Zealand [7]. A study on faecal samples collected from abattoirs showed a relatively high prevalence of neomycin resistance (38–67%) in *E. coli* in The Netherlands and a lower prevalence (17%) in Sweden [9]. Due to the presence of neomycin-resistant *E. coli* in pigs, it is advisable to perform antimicrobial susceptibility testing before treatment [2].

Despite the common use of neomycin in pig production, information from clinical trials on clinical and microbial recovery and the development of antimicrobial resistance when administering neomycin to newly weaned pigs with *ETEC*-related PWD is lacking. Due to the concentration-dependant nature of neomycin, we aimed to evaluate whether a single high dose would be effective in reducing the number of pigs with PWD compared to a standard dose and frequency, thus making it possible to reduce the use of antimicrobials for this disease. Furthermore, we aimed to evaluate the development of antimicrobial resistance following neomycin treatment.

## Materials and methods

### Study design

This study was performed as a randomised controlled clinical trial with two parallel groups of nursery pigs (Duroc x (Danish Landrace x Danish Yorkshire)) in January and February 2021 in a Danish conventional pig herd (with 535 sows) not using medicinal zinc in the starter diet. The herd had a history of diarrhoea outbreaks starting approximately 4 days post-weaning, and previous analysis showed that enterotoxigenic *E. coli* (ETEC) was highly prevalent among the newly weaned pigs. A previous screening from December 2018 revealed no neomycin resistance in the ETEC isolates collected from newly weaned pigs in the herd.

### Sample size considerations

We wanted to test for equality in cure rate between groups. The expected cure rate in the control group was 0.800. The null hypothesis was an equal cure rate (0.800) in the single high-dose group and the alternative hypothesis a cure rate of 0.700 in the single high-dose group. The test statistic used to calculate the sample size was the one-sided Z-test with pooled variance. The significance level of the test was targeted at 0.017, but the significance level actually achieved by this design was not known. To detect a difference of 0.100 between groups with 80% power, we need a group sample size of 258 in the single high-dose group and 439 in the control group.

### Treatment groups

In this study, the control group received a daily neomycin dose of 25,000 IU/kg bodyweight for 3 consecutive days as recommended by the supplier (Neomay, ScanVet Animal Health A/S), while the experimental group received a single high neomycin dose of 50,000 IU/kg bodyweight. For the control group, 10 g of neomycin sulphate powder was weighed in a measuring cup and drinking water was added up to the 40 ml mark. The solution was carefully mixed until all powder had dissolved. For the single high-dose group, the same procedure was followed using 20 g of neomycin sulphate powder. All animals were dosed according to bodyweight (0.1 ml neomycin sulphate solution per 0.5 kg bodyweight) at inclusion and the neomycin was administered individually and orally using a disposable 2.5 ml syringe without a needle.

### Sampling and treatment strategies

Newly weaned pigs were moved to nursery units on Thursdays, and the following Monday, Tuesday and Wednesday (days 4–6 after weaning), the pigs from that batch were included in the study. Pigs with perianal faecal staining were spray-marked on the back and examined for diarrhoea. Pigs with a faecal score of 3–4 [10] (classified as diarrhoea) were ear-tagged and weighed, and a clinical examination, rectal swab and faecal sampling were performed and neomycin treatment was administered. The single high-dose group received only neomycin treatment on the day of inclusion, but the control group was treated again on the following 2 days (3 days in total). All pigs were monitored daily for any adverse effects and a clinical examination, faecal sampling and rectal swab were repeated 4 days after the start of treatment (referred to as ‘after treatment’). Rectal swabs were stored for up to 2 days at 5℃ before microbiological analysis. Faecal samples were stored for up to 2 days at 5℃ before neomycin susceptibility analysis and then frozen at − 20℃ until dry matter analysis. All diarrhoeic pigs with faecal staining from six batches were sampled, and faecal scores were given by the same observer throughout the study. Pens were randomly allocated to either the control or single high-dose group, so all pigs included from one pen belonged to the same treatment group. Treatment groups were allocated in the following order: control, single high-dose, control, single high-dose, control, control, single high-dose, and this was repeated until all pens had been allocated a group. When allocating the first pen in the first batch to either the single high-dose or the control treatment group, the starting point was chosen at random by drawing lots.

### Faecal score and faecal dry matter analysis

Faecal samples were visually scored on a scale of 1 to 4. Faecal scores of 1–2 were considered normal faecal consistency and scores of 3–4 were considered to be diarrhoea, as described by Pedersen and Toft, 2011 [10]. Faecal dry matter was analysed to confirm whether the sample was diarrhoea or normal faeces. Empty containers were ID-marked and weighed on an analytical balance weight. Faecal samples were then thawed and homogenised before being weighed, and 1 to 3 g of sample material was used for the analysis [11]. Samples were dried for 12–18 h at 75℃ in a Mettler Toledo AE 240 Analytical Balance oven and then transferred to desiccators before being weighed. Dried samples were weighed and the faecal dry matter percentages were calculated using the formula: faecal dry matter percent % = ((faecal dry weight in container – weight of container)/faecal wet weight) × 100. A faecal dry matter content ≤ 18% was considered to be diarrhoea and > 18% was considered to be normal faeces [11].

### Bacteriology and polymerase chain reaction

Rectal swabs were cultured on blood agar plates for semi-quantitative counting (0%, 10%, 20%…100%) of haemolytic and non-haemolytic *E. coli*-like colonies, and polymerase chain reaction (PCR) analysis was used on a subsample of haemolytic *E. coli* isolates. Rectal swabs were used for the initial strokes (18 strokes) on blood agar plates and rotated during the process to ensure that there was enough sample material on the plate. The secondary strokes were performed with a 10 µl inoculation needle and touched the initial strokes 4–5 times. The third strokes were performed with a new 10 µl inoculation needle and touched the secondary strokes 1–2 times. The blood agar plates were incubated upside-down and overnight at 37℃. To ensure a consistent result, all plates were read by the same laboratory technician, blinded to treatment group. PCR analysis for F4, F18, STa, STb and LT was performed on a haemolytic isolate from a subgroup of pigs with ≥ 60% haemolytic *E. coli*-like colonies on the blood agar. If isolates were negative for both toxins and fimbria, a PCR was performed to confirm that they were *E. coli*. The multiplex PCR and virulence gene primers were used as described by Zhang et al. [12]. In brief, a single colony grown overnight was suspended in 50 µm of water. Bacterial suspensions were then boiled for 10 min, incubated on ice for 10 min, briefly centrifuged at maximum speed to pellet bacterial debris, and the supernatant was used as a DNA template. An initial heat activation step completed the multiplex PCR and the products were separated on 3% agarose gel. The *E. coli*-specific primers used have previously been described by Chen and Griffiths 1998 [13].

### Neomycin susceptibility testing

Faecal samples from approximately 20% of the pigs in each group (100 pigs in the control group and 68 pigs in the single high-dose) were used for neomycin susceptibility testing to compare neomycin resistance before and after treatment in both groups. The pigs were randomly selected from each group in each batch using an online random number generator [14] before susceptibility testing was performed using the method described below, which is a modified version of the test described by Græsbøll et al. in 2017 [15]. We suspended 1 g of faeces in 9 ml of phosphate buffered saline (PBS) to achieve a 10^–1^ dilution before using 1 ml of the initial suspension to make tenfold dilutions up to 10^–6^. From the 10^–2^ to 10^–6^ dilutions, 20 µl was placed on MacConkey agar plates (4 × 4 grid) with and without neomycin (16 mg/l) and incubated upside-down and overnight at 37℃. The neomycin concentration was based on the EUCAST epidemiological cut-off values for *E. coli* [16]. The following day, separated dark red colonies (> 0.5 mm) in the highest dilution were counted to calculate the colony forming units (CFU)/g faeces. All plates were read by the same laboratory technician, who was blinded to treatment group.

### Statistical analysis

Data analyses were performed using R version 4.1.0 (2021–05-18) [17] and statistical significance was accepted at P < 0.05. The tidyverse, rstatix and ggpubr packages were used for data management and analysis. The individual pig was the experimental unit. Descriptive analysis was performed using summary statistics. A logistic regression model with batch and pen as random effects was used to evaluate cure rates. Welch’s t-test was used to test the difference in mean CFU/g faeces on MacConkey agar plates with and without neomycin between groups as well as the difference in the mean percentage of haemolytic colonies and mean percentage of non-haemolytic colonies on blood agar plates between groups. Sensitivity, specificity and accuracy were calculated for the dichotomised faecal score (diarrhoea: 3–4, normal faeces: 1–2) as a measure of faecal dry matter content (≤ 18% or > 18%).

## Results

In total, 1,875 pigs from six batches (219–350 pigs per batch) were observed for perianal faecal staining and stained pigs were subsequently examined to determine a faecal score, with scores of 3–4 classified as diarrhoea. The apparent prevalence of diarrhoea on the first day of inclusion across all batches (n = 1,875) was 27% (n = 514), and a total of 802 pigs were sampled throughout the study. A total of 30 pigs were subsequently excluded due to other diseases requiring treatment, administration of the wrong dose, faecal scores below 3 or death. One pig died during the study, but this was not related to the neomycin treatment. After exclusion, 471 pigs remained in the control group and 301 in the single high-dose group. The percentage of included pigs ranged between 31 and 56% across the batches. The included pigs weighed on average 6.3 kg (standard deviation (sd) = 1.7) at the time of inclusion and treatment start. Some pigs presented with other clinical observations before onset of treatment, including ear wounds (22%), poor body condition (19%), long, dull and/or rough coat (8%), wounds (3%), umbilical or inguinal hernia (2%), joint thickening (2%), rectal hyperaemia (1%) and tail biting (< 1%). No adverse effects were observed in either group after the administration of neomycin.

### Treatment efficacy

Based on clinical faecal scores, the cure rates were 83% and 87% in the control and single high-dose group, respectively, with no statistically significant difference between groups (*p* = *0.174*). After correcting for faecal dry matter by excluding pigs with dry matter of > 18% at inclusion (n = 13) and all pigs for which dry matter analysis was not available, 397 pigs remained in the control group and 265 pigs remained in the single high-dose group. The cure rates based on dry matter analysis were 86% and 91% for the control and single high-dose groups, respectively, with a statistically significant difference between groups (*p* = *0.043*).

### Faecal dry matter analysis

Before treatment, 675 pigs had both a faecal score of 3 or 4 (representing clinical diarrhoea) and a faecal dry matter analysis. Less than 2% (n = 13) of these pigs had a faecal dry matter percentage above 18%, which was the cut-off for diarrhoea. The sensitivity, specificity and accuracy for dichotomised faecal scores (diarrhoea: 3–4, normal faeces: 1–2) as a measure of faecal dry matter content ≤ 18% or > 18% were 99%, 94% and 96%, respectively.

### Haemolytic and non-haemolytic E. coli-like colonies

**Fig. 1:**
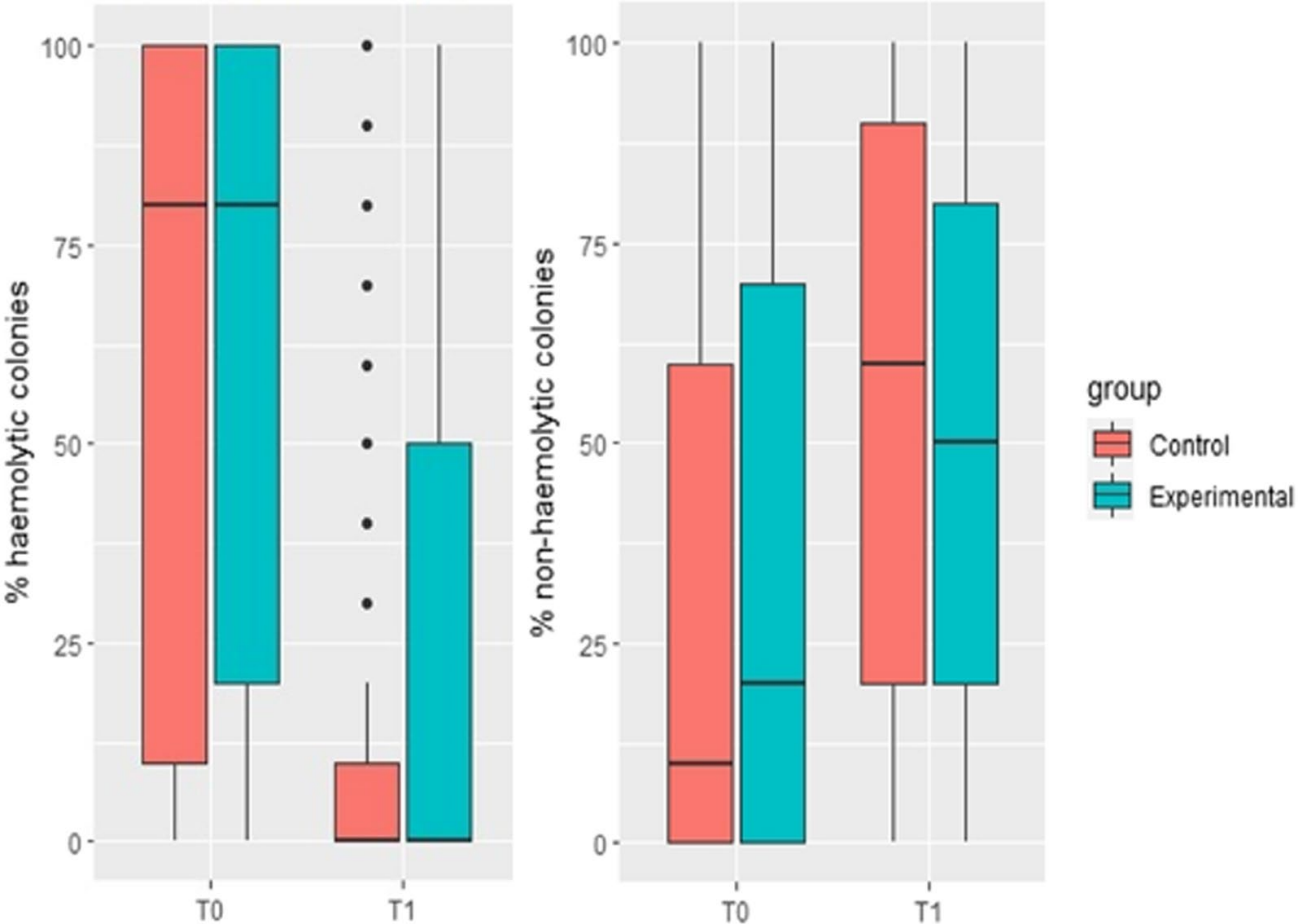
Haemolytic and non-haemolytic *E. coli-*like colonies. Boxplots showing the distribution of the percentage of haemolytic and non-haemolytic *E. coli-*like colonies on blood agar plates before (T0) and after (T1) neomycin treatment. Control group: 25,000 IU/kg neomycin for 3 consecutive days. Experimental/single high-dose group: Single neomycin dose of 50,000 IU/kg

Before treatment, no difference in the percentage of haemolytic and non-haemolytic *E. coli*-like colonies was found between groups (Table 1). After treatment, however, we found a significantly higher percentage of haemolytic *E. coli*-like colonies in the single high-dose group compared to the control group (*p* < *0.001*). This difference is also visualised in Fig. 1 and reflected in the number of pigs with ≥ 60% haemolytic *E. coli*-like colonies after treatment, with 24% in the single high-dose group compared to 9% in the control group (Table 2).

**Table 1:** Haemolytic and non-haemolytic *E. coli*-like colonies on blood agar before and after neomycin treatment

	Before treatment			After treatment		
	Control (n = 471)	Single high-dose (n = 301)	*P* value	Control (n = 471)	Single high-dose (n = 301)	*P* value
Mean (sd) percentage of haemolytic *E. coli*-like colonies	61% (41)	60% (39)	0.766	12% (26)	26% (37)	< 0.001
Mean (sd) percentage of non-haemolytic *E. coli*-like colonies	32% (37)	35% (37)	0.247	54% (35)	52% (35)	0.340

**Table 2:** Samples with ≥ 60% haemolytic *E. coli*-like colonies before and after neomycin treatment

	Before treatment		After treatment	
	Control (n = 471)	Single high-dose (n = 301)	Control (n = 471)	Single high-dose (n = 301)
≥ 60% haemolytic *E. coli*-like colonies	63% (n = 297)	61% (n = 183)	9% (n = 43)	24% (n = 73)

### PCR results

Single haemolytic colonies were sub-cultured from a randomly selected subgroup of samples with ≥ 60% haemolytic *E. coli*-like colonies and analysed using PCR. Before neomycin treatment, all analysed haemolytic isolates were positive for fimbria and toxins (ETEC positive), as shown in Table 3. Two different pathotypes were found in both the control and the single high-dose group, with F18 + STb + LT as the most frequent type. After treatment, 91% (n = 69) of all analysed isolates were positive for fimbria and one or more toxins in both the control and single high-dose group. Few isolates were negative for toxins and/or fimbriae, however all were identified as *E. coli* by PCR.

**Table 3:** Distribution of fimbria and toxins among samples with ≥ 60% haemolytic *E. coli*-like colonies before and after neomycin treatment

	Before treatment		After treatment	
	Control (n = 75)	Single high-dose (n = 67)	Control (n = 25)	Single high-dose (n = 51)
F18 + STb + LT	97% (n = 73)	97% (n = 65)	84% (n = 21)	75% (n = 38)
F4 + STb + LT	3% (n = 2)	3% (n = 2)	0%	6% (n = 3)
F18 + LT	0%	0%	8% (n = 2)	10% (n = 5)
F18^a^	0%	0%	8% (n = 2)	6% (n = 3)
*E. coli*^b^	0%	0%	0%	4% (n = 2)

^a^Toxin negative

^b^Fimbria and toxin negative

### Neomycin susceptibility testing

**Fig. 2:**
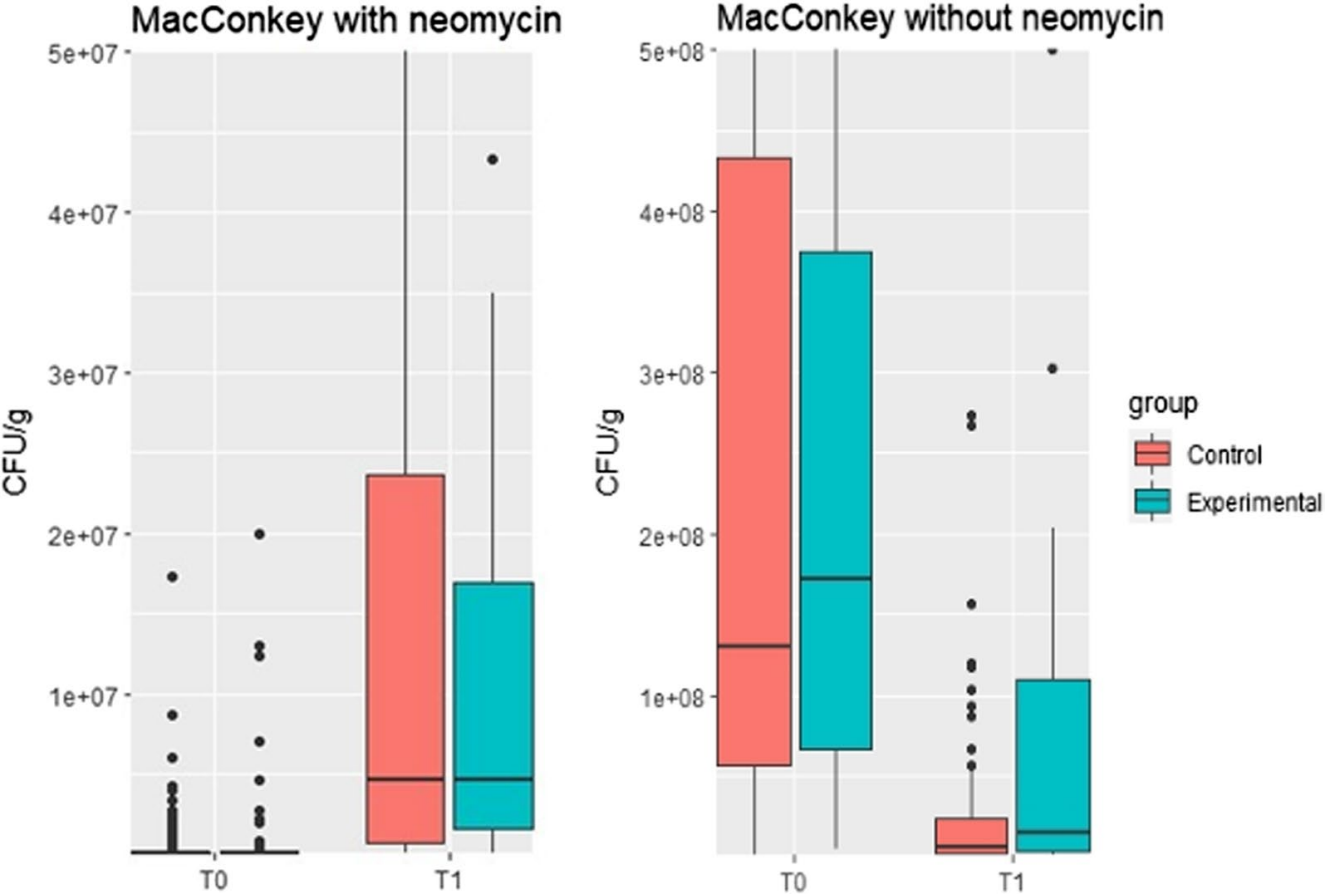
CFU/g faeces on MacConkey agar. Boxplots showing the distribution of CFU/g faeces before (T0) and after (T1) treatment on MacConkey agar plates with and without neomycin (16 mg/l). Note the difference in the y-axis. Control group: 25,000 IU/kg neomycin for 3 consecutive days. Experimental/single high-dose group: Single neomycin dose of 50,000 IU/kg

Figure 2 shows the CFU/g faeces on selective MacConkey agar plates with and without neomycin. Without neomycin, a drop in CFU/g after treatment was seen in both groups, indicating a reduction in the total number of Enterobacteriaceae. The selection of resistant bacteria after antimicrobial treatment with neomycin was observed as an increase in CFU/g after treatment. There was no statistically significant difference between groups with and without neomycin, either before or after treatment (Table 4).

**Table 4:** CFU/g faeces on selective MacConkey agar plates with or without neomycin (16 mg/l) before and after neomycin treatment

	Before treatment			After treatment		
	Control (n = 100)	Single high-dose (n = 68)	*p* value	Control (n = 100)	Single high-dose (n = 68)	*p* value
Mean (sd) CFU/g on plates without neomycin	3.3 × 10^8^ (4.4 × 10^8^)	3.4 × 10^8^ (4.9 × 10^8^)	0.877	7.9 × 10^7^ (3.0 × 10^8^)	1.6 × 10^8^ (3.6 × 10^8^)	0.120
Reduction in CFU/g				− 2.5 × 10^8^	− 1.8 × 10^8^	
Mean (sd) CFU/g on plates with neomycin	4.1 × 10^6^ (2.6 × 10^7^)	1.0 × 10^6^ (3.3 × 10^6^)	0.251	6.5 × 10^7^ (2.4 × 10^8^)	4.8 × 10^7^ (1.3 × 10^8^)	0.548
Increase in CFU/g				+ 6.1 × 10^7^	+ 4.7 × 10^7^	

## Discussion

In this study, nursery pigs with ETEC-related PWD from six batches in a Danish herd were treated orally and individually with neomycin either once at a dose of 50,000 IU/kg bodyweight (single high-dose group) or on 3 consecutive days at a dose of 25,000 IU/kg bodyweight (control group). Neomycin is commonly administered through the water to entire pens or batches of nursery pigs during an outbreak of diarrhoea, thus both diarrhoeic and non-diarrhoeic pigs are treated [18]. Furthermore, there is a chance that some pigs may not receive the advised dosage due to a lower-than-expected water uptake. This study showed that individual oral treatment with a single high dose (50,000 IU/kg) of neomycin is effective in reducing the number of pigs with ETEC-related PWD, with a cure rate of 87% (91% when correcting for faecal dry matter) in the single high-dose group. This cure rate (87%, n = 301) was comparable to that of the control group (83%, n = 471) based on faecal scores, and significantly higher (91%, n = 265 *vs.* 86%, n = 397) when based on faecal dry matter analysis (*p* = *0.043*). This shows that the concentration-dependant properties of neomycin can be used in neomycin treatment protocols for ETEC-related PWD and can contribute to a reduction in the total amount of neomycin used per pig. Administering a single high dose of neomycin can result in a 33% reduction in the total amount of antimicrobial used when compared to standard treatment protocols. To the authors’ knowledge, this has not previously been reported. Furthermore, individual treatment with neomycin is advantageous because it is easier to ensure that each animal receives the correct dose when compared to batch medication [19]. Several other studies on aminoglycoside use in both humans and animals show that a high initial dose and minimum 24-h intervals are preferable [20–22]. A high initial dose may also result in a longer post antimicrobial effect. Because of the labour intensiveness of treating nursery pigs individually, an individual oral administration of neomycin could be a challenge in some herds, especially at larger outbreaks. It would be relevant to evaluate if the single high-dose treatment regimen can be used for water medication in herds with major outbreaks of PWD, where individual treatment is not an option. However, if the pigs are sorted by weight at weaning and housed accordingly, a drench would be a relevant tool for easy administration of the neomycin solution. When treating individually, the single high-dose treatment regimen has the advantage that it is less stressful for the pigs than being handled on three consecutive days.

Many of the clinical observations in this study – such as poor body condition and altered coat appearance – are commonly observed in pigs with PWD. However, ear wounds may result from the pigs mixing after weaning.

The mean percentage of haemolytic *E. coli*-like colonies was significantly higher in the single high-dose group after treatment with neomycin. One explanation for this may be the difference in time between the last neomycin dose and sampling after treatment, but it could also be an indication of the standard treatment being more effective in reducing the number of haemolytic bacteria in the faeces. The assessment of colonies was semi-quantitative, but all plates were read by the same laboratory technician blinded to the treatment group. It is unknown whether the slight difference in sample storage time would have an impact on *E. coli* cultivation. The higher number of haemolytic *E. coli*-like colonies in the single high-dose group after treatment might indicate an increased risk of relapse, however this was beyond the scope of this study. All PCR-analysed haemolytic isolates sampled before neomycin treatment were positive for fimbria (either F4 or F18) and toxins (STb and LT), which emphasises that the PWD outbreaks in the six batches were indeed ETEC-related, with F18 + STb + LT being the most frequent type. We found no evidence of increased neomycin resistance in coliforms in the single high-dose group compared to the standard group. The antimicrobial mantra “shorter is better” is increasingly used in human medicine; short-course treatments have been compared to traditional courses of antimicrobial therapy in systematic reviews of several randomised controlled trials, with no difference in efficacy found between the treatments [23, 24]. However, the overuse of antimicrobials will drive the selective pressure for antimicrobial resistance.

Accurate diagnosis and antimicrobial susceptibility tests are crucial when initiating treatment against PWD, and other measures to reduce the transmission of pathogenic bacteria must also be implemented. Although this study was conducted in one Danish pig herd, it is plausible that the results can be extrapolated to other nursery pig herds experiencing similar outbreaks of ETEC-related PWD.

## Conclusion

A comparable or higher recovery rate in the single high-dose group suggests that a single high dose (50,000 IU/kg) of neomycin can effectively treat ETEC-related PWD and reduce antimicrobial use by 33% compared to three standard treatment doses (25,000 IU/kg for 3 consecutive days). The study showed a higher number of haemolytic *E. coli* in the single high-dose group after treatment, but no evidence of increased neomycin resistance in coliforms was observed for the single high-dose treatment compared to standard treatment.

## Data Availability

Dataset is available from the corresponding author upon reasonable request.

## Abbreviations

CFU: Colony forming units
ETEC: Enterotoxigenic *Escherichia coli*
PCR: Polymerase chain reaction
PWD: Post-weaning diarrhoea
